# Editorial: Selenium and Selenoproteins in Brain Development, Function, and Disease

**DOI:** 10.3389/fnins.2021.821140

**Published:** 2022-01-13

**Authors:** Matthew W. Pitts, Peter R. Hoffmann, Lutz Schomburg

**Affiliations:** ^1^Department of Cell and Molecular Biology, University of Hawaii at Manoa, Honolulu, HI, United States; ^2^Institute for Experimental Endocrinology, Charité University Medicine Berlin, Berlin, Germany

**Keywords:** selenium, selenoprotein, brain, neurodevelopment, neurodegeneration, oxidative stress

Selenium (Se) is an essential micronutrient with important effects on the brain and cells of the nervous system. Its influence is mediated primarily through selenoproteins, a class of proteins characterized by the co-translational incorporation of Se as the amino acid selenocysteine. These proteins play fundamental roles in redox signaling, protection from damage, endocrine homeostasis etc. and include the glutathione peroxidases, thioredoxin reductases, and iodothyronine deiodinases. The human genome encodes 25 distinct selenoproteins, along with a host of additional Se-related proteins involved in selenoprotein biosynthesis and Se metabolism. Many of these are highly expressed in brain, and mouse knockout studies have shown that several are indispensable for neurodevelopment and protection from neuronal damage, as e.g., shown for parvalbumin-expressing interneurons, a class of GABAergic neurons characterized by high rates of metabolism. Humans with rare mutations in selenoprotein biosynthesis genes exhibit neurological defects that parallel those detailed in knockout mice, including deficits in cognition and motor function, seizures, hearing loss, and altered thyroid metabolism.

The goal of this Research Topic was to assemble a collection of state-of-the-art articles pertaining to the influence of selenium (Se) and / or selenoproteins on brain development, function, and disease. This resulted in a compilation of four original research and five review articles from Se researchers around the globe.

Several of manuscripts were devoted to the basic science of Se /selenoproteins. Schweizer et al. provide a concise overview of the various roles of individual selenoproteins in brain, along with associated Se-related proteins involved in selenoprotein biosynthesis. These authors also offer food for thought regarding issues that remain unsolved in Se biology. Solovyev et al. present an in-depth review of Se transport and homeostasis at the blood-brain barrier, a matter of great importance to maintenance of proper redox balance in brain. The relationship between stress and selenium homeostasis in brain is explored by Torres, Alfulaij et al. with thorough review of published studies employing selenocompounds in rodent models of stress. Martinez and Hernandez provide newfound insight into the developmental regulation of brain thyroid metabolism, showing that type 3 deiodinase is a critical negative regulator of thyroid hormone action during the fetal period. Also, in an original research report, Kilonzo et al. assess the developmental effects of varying levels of Se supplementation upon neurobehavioral and metabolic indices in adulthood, detailing that Se-deficiency leads to deficits in cognition, altered sensorimotor gating, and increased adiposity. Finally, Torres, Yorgason et al. supply first evidence of a modulatory role for Selenoprotein P in mesolimbic dopaminergic signaling.

The remaining publications in this collection focus upon the roles of Se / selenoproteins in various disease states. A clinical study conducted by Seelig et al. identify the enigmatic Selenium-Binding Protein 1 as a biomarker for adverse outcomes following traumatic spinal cord injury. Notably, this work builds upon earlier findings of elevated SELENBP1 levels in the context of neuropsychiatric disease (Glatt et al., 2005; Udawela et al., 2015), and the recent demonstration of SELENBP1 as a methanethiol oxidase (Pol et al., 2018). An overview of the potential therapeutic usage of Se in the treatment of glioblastoma is presented by Yakubov et al., with specific focus upon brain edema, glioma-related angiogenesis, and glioma-associated microglia. Lastly, the important topic of Se and Alzheimer's disease (AD) is covered by Zhang and Song. The authors detail links between known functions of individual selenoproteins and pathological alterations in AD, such as elevated endoplasmic reticulum stress, impaired calcium homeostasis, and neuroinflammation.

Collectively, the publications indicate the grown maturity of our knowledge on the essential roles of Se and selenoproteins for brain development and protection from neuronal loss. The wide spectrum of aspects covered by this Research Topic nicely mirrors the expanding understanding of the diverse roles played by the different selenoproteins along with the new perspectives for taking advantage of these insights in the quest for nutritional and therapeutic support in the preservation of our sensory and intellectual functions. We are convinced that the articles published in this Research Topic contribute to this important aim and provide the readership with essential knowledge, stimulating thoughts, and strong motivation along this line.

## Author Contributions

All authors listed have made a substantial, direct, and intellectual contribution to the work and approved it for publication.

## Conflict of Interest

LS holds shares in selenOmed GmbH, a company involved in selenium status assessment and supplementation. The remaining authors declare that the research was conducted in the absence of any commercial or financial relationships that could be construed as a potential conflict of interest.

## Publisher's Note

All claims expressed in this article are solely those of the authors and do not necessarily represent those of their affiliated organizations, or those of the publisher, the editors and the reviewers. Any product that may be evaluated in this article, or claim that may be made by its manufacturer, is not guaranteed or endorsed by the publisher.
